# Systematic use of long-acting intramuscular progesterone in addition to oral dydrogesterone as luteal phase support for single fresh blastocyst transfer: A pilot study

**DOI:** 10.3389/fendo.2022.1039579

**Published:** 2022-12-23

**Authors:** Virginie Simon, Geoffroy Robin, Laura Keller, Camille Ternynck, Sophie Jonard, Camille Robin, Christine Decanter, Pauline Plouvier

**Affiliations:** ^1^ Department of Assisted Reproductive Technologies and Fertility Preservation, Jeanne de Flandre Hospital, Lille, France; ^2^ Univ. Lille, Faculty of Medicine, Lille, France; ^3^ Institut de Biologie de la Reproduction-Spermiologie-Centre d'étude et de Conservation des Oeufs et du Sperme Humain (CECOS), Hôpital Jeanne de Flandre, Centre Hospitalier Universitaire de Lille, Lille, France; ^4^ Univ. Lille, University Hospital Center (CHU) Lille, Research Unity (ULR) 2694-METRICS: Evaluation des Technologies de Santé et des Pratiques médicales, Lille, France; ^5^ University Hospital Center (CHU) Lille, Department of Biostatistics, Lille, France

**Keywords:** luteal phase support, dydrogesterone, pregnancy rates, IVF, intramuscular progesterone

## Abstract

**Objective:**

The need of luteal support after FET is no longer to be proven. Different routes of progesterone administration are available with interindividual differences in metabolization and serum progesterone levels, the latter being highly correlated with pregnancy and delivery rates. The administration of 2 different routes of progestogen significantly improves success rates in FET. The aim of the current study was to investigate the added value to combine intramuscular administration of progesterone to dydrogesterone in fresh embryo transfer.

**Methods:**

This is a retrospective study from prospectively collected data. Patient, aged between 18 and 43 years old, had received a fresh blastocyst transfer between January 2021 and June 2021. In the first group, all patients received only oral dydrogesterone 10mg, three times a day, beginning the evening of oocyte retrieval. In the second group, patients received, in addition to dydrogesterone, a weekly intramuscular injection of progesterone started the day of embryo transfer. Primary endpoint was ongoing pregnancy rate.

**Results:**

171 fresh single blastocyst transfers have been performed during this period. 82 patients were included in “dydrogesterone only” and 89 patients in “dydrogesterone + IM”. Our two groups were comparable except for body mass index. After adjustment on BMI, our two groups were comparable regarding implantation rate, early pregnancy rate (46.1 versus 54.9, OR 1.44 [0.78; 2.67], p=0.25) miscarriage rate, ongoing pregnancy rate (30.3 versus 43.9, OR 1.85 [0.97; 3.53] p= 0.06).

**Conclusion:**

Using systematically long acting intramuscular progesterone injection in addition to oral dydrogesterone as luteal phase support seems to have no significant impact on IVF outcomes when a single fresh blastocyst transfer is performed.

## Introduction

During IVF treatment, patients show a lack of progesterone secretion after oocyte retrieval. There are many reasons to explain this phenomenon of which the increase in steroid levels during COS secreted by growing follicles, resulting in a negative feedback of LH secretion during luteal phase and a premature luteolysis (1–3).

Progesterone plays a key role in the establishment and maintenance of pregnancy. The need of a luteal phase support in IVF is now well known, but its modalities are still debated (administration routes, duration of administration and time to initiate the treatment) (4–8).

Different routes of administration exist: vaginal, oral, intramuscular (IM), subcutaneous (SC) and even rectal route. Use of vaginal progesterone seems to be the « gold standard » (9).

Numerous studies have confirmed the equivalent effectiveness of the different routes of administration of progesterone (except oral micronized progesterone, which is not effective) (6, 10–15). A recent meta-analysis and review showed that vaginal and IM administration were the most commonly used (16).

We know, especially from studies analyzing progesterone thresholds and their effects on pregnancy outcomes in artificial cycles for frozen-thawed embryo transfer, that low progesterone rates affect pregnancy outcomes and that some patients may suffer from sub-optimal luteal phase support (17–19). These findings may support the idea of developing rescue strategies using for example additional subcutaneous progesterone injection (20).

Some authors have also experimented the administration of two routes of progesterone; weekly intramuscular progesterone in addition to a classic luteal phase supported by vaginal progesterone versus vaginal progesterone alone with similar results between the two treatments after fresh embryo transfer (21).

Despite equivalent dose and route of administration of progesterone, high differences of progesterone levels have been observed from one patient to another, with adverse effects on success rates (22, 23). Inter- and intra-individual variations also exist considering progesterone bioavailability. On the contrary, too much progesterone could lower the pregnancy rates (24, 25). Indeed, high levels of progesterone may also interfere with embryo implantation by affecting the endometrial implantation window and have a deleterious effect, as shown in a recent study which explored serum progesterone levels in early and mid-luteal phases in IVF (22).

In fact, additional luteal phase support may rescue endometrial maturation in some patients but may have a deleterious effect in others.

Dydrogesterone is a progestin that has a non-planar three-dimensional structure and thus belongs to the retroprogesterone family. In contrast to progesterone, dydrogesterone has a higher oral bioavailability (26, 27) and induces endometrial transformation at a dose 10 to 20 times lower than that of micronized progesterone (28, 29). Its plasma half-life is approximately 5-7 hours. Due to its chemical structure, this progestin binds almost exclusively to progesterone receptors with a pure progestational activity (29, 30).

The LOTUS trial highlighted the non-inferiority of luteal phase support with oral dydrogesterone in comparison to vaginal micronized progesterone in terms of live birth rates (31–34), confirming that oral dydrogesterone (DYD) provide at least similar reproductive outcomes than vaginal progesterone as showed before (35). More recently, two French cohort studies showed similar results (36, 37). In addition, oral luteal phase support with dydrogesterone may be better tolerated by patients (33). So, dydrogesterone seems to be a safe and effective choice to support luteal phases in IVF. However, in the two recent real-life studies of IVF world published in 2020 and 2021 on current IVF practices, the authors show that the majority of centers still use the vaginal route (38, 39).

Despite its apparent simplicity and effectiveness, the hepatic metabolism of oral dydrogesterone is complex. It induces the formation of 45 different metabolites (40–42). It is therefore not impossible that variations in the metabolism of this progestogen (conditioned by genetic polymorphisms in liver enzymes) may modulate its efficacy as a progestogen, as is found for many drugs (pharmacogenetics). A recent study on artificial cycles for frozen-thawed embryo transfers showed inter- and intra-individual variations of DYD and one of its active metabolites, independently of BMI and body weight (43). Furthermore, even if the oral route is more comfortable for patients, compliance problems cannot be formally excluded: for example, forgetting to take dydrogesterone tablets that should ideally be administered three times a day.

Thus, in order to compensate for these possible disadvantages of dydrogesterone (which have not yet been studied), we compared two different luteal phase supports in terms of ongoing pregnancy after a single fresh blastocyst transfer: dydrogesterone alone versus the use of two systematic routes of progesterone luteal phase supports including dydrogesterone and an additional long-acting intramuscular injection of 500 mg of progesterone.

## Material and methods

This is a retrospective analysis of prospective data collected in the French national ART database named “JFIV” performed in the ART Department of Lille University Hospital from January 2021 to June 2021 based on an historic cohort. All patients had given prior consent for the use of their clinical, hormonal and ultrasound data. On December 16, 2019, the Institutional Review Board of the Lille University Hospital gave unrestricted approval for the anonymous use of all patients’ clinical, hormonal and ultrasound records (reference DEC20150715-0002).

Patients included in this study were aged between 18 and 43 years and had received a fresh embryo transfer of a blastocyst stage embryo between January 2021 and June 2021.

We excluded:

Patients who received a transfer of two embryos (early cleaved stage and blastocyst stage)

Patients who received a fresh embryo transfer at the early cleaved stage

Oocyte recipient patients

No fresh embryo transfer (freeze-all, elevated progesterone level, inadequate endometrium)

Patients receiving special luteal phase treatment (after immunological tests for example)

### COH protocol

The ovarian stimulation protocol (short GnRH antagonist or long GnRH agonist protocol) and the type of gonadotropin (HMG or recombinant FSH alone) and FSH starting dose was chosen by the referring physician in consultation, according to age, BMI, AMH and AFC.

Ovulation triggering was decided when at least 3 follicles with an average diameter strictly greater than 17 mm were visualized in ultrasonography examination. The U/S examination was performed using a Voluson E8 Expert (General Electric Systems, VELIZY, France) and a 5-9 MHz transvaginal transducter. Ovulation was triggered by an injection of Ovitrelle^®^ (Choriogonadotropin alpha, 250 μg, Merck Serono, Lyon, France). Oocytes were retrieved (using transvaginal ultrasound-guided needle aspiration) 36 h after the hCG injection.

### Luteal phase support

From January 2021 to the 15th of April 2021, all patients received only oral dydrogesterone 10mg, three times a day, as luteal phase support beginning the evening of oocyte retrieval.

Dydrogesterone was continued until the pregnancy test performed twelve days after the embryo transfer. If the test was positive, patients were told to continue dydrogesterone until 6 weeks of gestation. This group was named “dydrogesterone only”.

The second group named “dydrogesterone + IM” (from 15th of April 2021 to June 2021) received oral dydrogesterone 10mg, three times a day, beginning the evening of oocyte retrieval. The day of fresh embryo transfer, patients received one supplementary long-acting intramuscular injection of progesterone (hydroxyprogesterone caproate, 500mg/2mL, Bayer HealthCare, Loos, France), Dydrogesterone was continued until the pregnancy test performed twelve days after the embryo transfer. If the test was positive, patients were told to continue only oral dydrogesterone until 6 weeks of gestation.

### Selection of blastocyst transferred

Retrieved oocytes were fertilized either by insemination (IVF) or by injection (ICSI) and incubated in a 6% CO2, 5% O2 controlled environment at 37°C (*ESCO^®^ Miri*). Fertilization was checked 16-18h after procedure. Blastocysts assessment was evaluated according to the Gardner classification at Day 5 (44). The morphologic criteria were based on degree of blastocoel cavity expansion, number, and cohesiveness of the inner cell mass (ICM) and trophectoderm (TE). Only fully expanded blastocysts (>=B3) with a ICM and TE grade A or B were eligible for potential transfer. The blastocyst with the highest score was transferred. All patient received a single blastocyst transfer.

### Transfer technique of embryos

Embryo transfer (ET) was performed with the Elliocath^®^ Angled Catheter (Ellios BioTek Laboratory, Paris, France). The catheter containing the embryo was introduced into the uterine cavity and the embryo was deposited at 1.5 - 2 cm from the uterine fundus according to ultrasound guidance. Subsequently, the catheter was immediately examined under the microscope to ensure that the embryo did not accidentally remain in the catheter.

### ET outcomes

A pregnancy test was performed by assaying plasma quantitative hCG twelve days post ET. A pregnancy was defined by hCG level higher than 100 mIU/mL. Ongoing pregnancy was defined as the pregnancy that progressed beyond 10 weeks of gestation. A spontaneous miscarriage was defined by the non-evolution of a pregnancy before 22 weeks of gestation; early if the miscarriage occurred before 10 weeks of gestation and late if it occurred between 10 and 22 weeks of gestation.

### Statistical analysis

Quantitative variables were expressed as means ± standard deviation (SD) or medians (interquartile range) for non-normal distributions, and categorical variables were expressed as numbers (percentage). Normality of distributions was assessed using histograms and tested using the Shapiro-Wilk test. Baseline characteristics were described and compared according to administration of intramuscular progesterone using the chi-square test (or Fisher’s exact test, when the expected cell frequency was < 5) for qualitative variables, and with Student’s t test (or a Mann-Whitney U test for variables with a non-Gaussian distribution) for quantitative variables.

The primary aim of the present study was to study the impact of the addition of intramuscular progesterone on early pregnancy rate. Secondary objectives were to study the impact of the administration of intramuscular progesterone on miscarriages, ongoing pregnancies, ectopic pregnancies, twin pregnancies, and implantation rate. The impact of intramuscular progesterone on these different events was studied using logistic regression models with and without adjustment on BMI. For each outcome, we examined the log-linearity assumptions for BMI using restricted cubic spline functions. Odds ratios (OR) and their 95% confidence intervals (95% CI) were calculated as effect size.

Statistical testing was done at the 2-tailed α-level of 0.05. Data were analyzed using SAS software version 9.4 (SAS Institute, Cary, NC).

## Results

171 fresh embryo transfers were performed and included in our study during this period.

Patients were divided into two groups, i.e., dydrogesterone –only and dydrogesterone + IM, the one having received the classic luteal phase support described before (dydrogesterone only, 89 patients) and the one having received a weekly IM progesterone injection in addition to the usual luteal phase support (dydrogesterone + IM, 82 patients).

Initial patients’ characteristics are described in **Table 1**.

**Table 1:** Patients characteristics.

single blastocyst transfer (N = 171)			
	No IM progesterone (N = 89)	IM progesterone (N = 82)	p-value
Age (years)	33.99 ± 4.71	33.70 ± 4.19	0.68
BMI (kg/m^2^)	23.84 ± 4.27	25.47 ± 5.20	**0.027**
Smoking	11/89 (12.4%)	8/81 (9.9%)	0.61
AMH (pmol/L)	20.7 [11.8; 29.6]	19.9 [14.3; 29.1]	0.80
**Infertility indication**			0.48
Idiopathic	16 (18%)	19 (23.2%)	
Tubal	8 (9%)	13 (15.9%)	
Endometriosis	16 (18%)	10 (12.2%)	
ICSI	41 (46.1%)	31 (37.8%)	
Ovulatory	5 (5.6%)	4 (4%)	
Multiple indications	3 (3.4%)	5 (6.1%)	
Rank attempt of IVF +/- ICSI	1 [1; 2]	1 [1; 2]	0.84

Values are expressed in number (%) or in median [interquartile range] or in mean ± standard deviation.

BMI, body mass index; ICSI, Intracytoplasmic Sperm Injection; AMH, Anti Mullerian Hormone; Access-dxi Beckman Coulter (USA).

Bold values denote statistical significance at the p < 0.05 level.

Our two groups were comparable for patient age, smoking status, ovarian reserve represented by AMH, infertility indication and the rank of attempt of IVF +/- ICSI except for body mass index.

Our two groups were comparable for all initial characteristics of IVF cycle (**Table 2**).

**Table 2:** Characteristics of IVF cycle.

Single blastocyst transfer (N = 171)			
	No IM progesterone (N = 89)	IM progesterone (N = 82)	p-value
**IVF technology:**			0.34
IVF	38 (42.7%)	41 (50%)	
ICSI	51 (57.3%)	41 (50%)	
**Ovary stimulation protocol**			0.60
Antagonist	42 (47.2%)	42 (48.8%)	
Agonist	47 (52.8%)	40 (51.2%)	
Duration of stimulation (days)	11 [10; 12]	11 [10; 12]	0.31
Total dose of FSH	2513 [1500; 3300]	1975 [1375; 3300]	0.19
Number of follicles >15 mm (trigger day)	9.55 ± 3.66	8.44 ± 3.92	0.057
Oocytes retrieved	8.89 ± 4.24	8.70 ± 4.70	0.78
Oocytes fertilized	7.62 ± 3.71	7.71 ± 4.18	0.88
Number of embryos obtained	5 [3; 7]	4.50 [3; 7]	0.51
Number of embryos vitrified	1 [0; 3]	1 [0; 3]	0.57

Values are expressed in number (%) or in median [interquartile range] or in mean ± standard deviation.

Our two groups were comparable regarding implantation rate, early pregnancy rate (46,1 versus 54,9, OR 1.44 [0.78; 2.67], p=0.25), miscarriage rate, ongoing pregnancy rate (30,3 versus 43,9, OR 1.85 [0.97; 3.53] p= 0,06) and ectopic pregnancy rate (**Table 3**).

**Table 3:** IVF attempt outcomes.

Single blastocyst transfer (N = 171)						
	No IM progesterone (N = 89)	IM progesterone (N = 82)	Odds ratio	p-value	Odds ratio adjusted	Adjusted p-value
Implantation rate	37.8% (42/90)	42.7% (35/82)	1.23 [0.66; 2.26]	0.51	1.26 [0.67; 2.36]	0.47
Early pregnancy rate	46.1% (41/89)	54.9% (45/82)	1.42 [0.78; 2.60]	0.25	1,44 [0.78; 2.67]	0.25
Miscarriage rate	24.4% (10/41)	13.3% (6/45)	0.48 [0.16; 1.46]	0.19	0.52 [0.17; 1.62]	0.26
Ongoing pregnancy rate	30.3% (27/89)	43.9% (36/82)	1.80 [0.96; 3.37]	0.067	1.85 [0.97; 3.53]	0.06
Ectopic pregnancy	9.8% (4/41)	6.7% (3/45)	0.66 [0.14; 3.15]	0.60	0.49 [0.09; 2.58]	0.41
Twin pregnancy rate	2.25% (2/89)	0% (0/82)	NA	NA	NA	NA

Results are expressed as percentage (number) or OR (95% CI) with and without adjustment for BMI and as unadjusted and adjusted P values. OR, odds ratio; CI, confidence interval.

NA, not applicable.

## Discussion

In our study, we did not observe a significant difference in pregnancy rates after single fresh blastocyst transfer after addition of an intramuscular progesterone injection (30.3% versus 43.9; OR 1.85 [0.97; 3.53] adjusted p= 0.06).

Vuong et al. explored recently the early luteal phase hormonal profile in patients undergoing ovarian stimulation in IVF after triggering by hCG and without any exogenous luteal phase support (3). The authors showed that serum progesterone level started to decrease five days after oocyte retrieval. A dramatic decline occurred four to six days after oocyte pick-up, which means the day of the fresh blastocyst transfer, when we decided to give an additional intramuscular progesterone injection in our study.

Some studies suggest tailoring luteal phase support modalities to each patient based on progesterone levels, particularly in hyperresponders in whom GnRH agonist induction is indicated, before adding or not adding a recombinant hCG injection (45). This is referred to as individualized phase support. From one patient to another, it seems that the need for progesterone levels differ. In a retrospective study of 1041 patients, the authors found significant efficacy of additional luteal phase support in patients with low mid-luteal phase progesterone when a fresh embryo transfer is performed (46).

Indeed, early luteal phase progesterone level (P4) appear to predict pregnancy outcomes as showed in a retrospective study published in 2019 when oral dydrogesterone is used for luteal phase support (23). Due to structural differences with progesterone, it remains difficult for dydrogesterone or its metabolites to compare progesterone measurements (27). The results of this study were completely opposite to a study published in 2018 (22). In this study, the authors showed a negative impact of very high level of P4 on pregnancy outcomes, but the luteal phase support used was vaginal progesterone.

These studies show that at equal luteal phase support, progesterone levels differ between patients and that this could have an impact on the pregnancy rates obtained after embryo transfer. However, it seems difficult in current practice to adapt the phase support to each patient.

A recent meta-analysis (19) showed a positive correlation between progesterone concentration and ongoing pregnancy rates when patients are divided by the pregnancy status but in this study there was no significant correlation between low progesterone levels and ongoing pregnancy rates when comparing low to high progesterone concentration groups.

The reason why these variations could have an impact on pregnancy outcomes is not really understood. As shown by Labarta et al. (47), it seems that there is no correlation between progesterone serum levels and progesterone and metabolites in the uterus nor between serum progesterone levels and endometrial receptivity.

Oral DYD has high bioavailability and shows a low hepatic first-pass effect (38).

A study looking at the use of dydrogesterone in artificial cycles for frozen-thawed embryo transfer (no endogenous progesterone effects) has recently shown inter- and intra-individual variations of serum levels of DYD and its active metabolite 20α-dihydrodydrogesterone (DHD). These variations were not explained by body mass index and body weight, and may influence pregnancy outcomes (43), as it has been published with micronized vaginal progesterone in artificial cycle (18). In these two studies, a minimum threshold was demonstrated, but not an upper one.

To our knowledge, this is the first study comparing DYD alone versus DYD and administration of one bolus of 500 mg of intramuscular progesterone as luteal phase support in IVF on pregnancy outcomes. Aqueous progesterone is available as a daily subcutaneous or intramuscular injection (25 mg/day) in France but is not reimbursed by the French health insurance system. Its cost is also high, which makes its prescription very limited. On the other hand, the weekly form of progesterone (500 mg/week) is reimbursed and has the advantage of optimising compliance due to its simplicity and long duration of action.

Intramuscular progesterone injection greatly increases P4 serum levels but has a moderate effect on uterine tissue concentration (48). This may explain why this luteal phase protocol doesn’t appear to have a deleterious effect. Indeed, too high progesterone levels in the uterine cavity could theoretically have induced a premature opening of the implantation window and thus a disruption of the physiological synchronicity between endometrial maturation and embryonic development. On the contrary, the systematic use of a bolus of 500 mg of intramuscular progesterone in addition to dydrogesterone may compensate for possible moderate pharmacokinetic variations or compliance problems of oral dydrogesterone and may have a beneficial effect on ongoing pregnancy rates after fresh embryo transfer. Indeed, in our study, we observed a non-significant difference of 13.3% on ongoing pregnancy rates in favor of the group that received intramuscular progesterone in addition to oral dydrogesterone and a decrease of almost 11% of miscarriage rates in favor of the group receiving additional intramuscular progesterone. The lack of significance of this result could perhaps be due to the small size of our sample, thus confirming the need for a larger randomized study.

Indeed, our pilot study has some weaknesses. The most important one is the size of our patient sample size. However, all patients performing a fresh blastocyst transfer during the inclusion period were included which limited selection bias. Due to the changes in the reproductive biology laboratory in our center (new equipment to offer optimal prolonged embryo culture up to the blastocyst stage) and the strong disruption of IVF activity at the beginning of the COVID-19 pandemic, we were not able to increase the sample size in order not to induce further bias. We lack of adequate statistical power to detect differences between the two groups since no formal sample size calculation was done. To detect a difference of 14% of ongoing pregnancy rate in balanced parallel groups trial, by two sided test at 0.05 significance level, with a power of 80%, we will need to include 197 patients per group (394 patients in total).

Prospective randomized studies are needed to confirm that using systematically two different routes of progesterone administration in a “combined strategy” for luteal phase support is not deleterious and could even improve pregnancy outcomes.

## Conclusion

In IVF, the need of luteal phase support is no longer to be proven after fresh embryo transfer. DYD seems to be a safe and effective choice as luteal phase support but inter- and intra-individual variations of progesterone serum levels of DYD exist and may influence pregnancy outcomes in some women. In our study, we investigated the impact of a systematic use of intramuscular progesterone in addition to oral dydrogesterone as luteal phase support, which may compensate for these variations, and eventual compliance problems. We did not find any significant difference between our two groups on pregnancy outcomes. Larger prospective randomized studies are needed to confirm these results and to analyze the potential benefit of such a therapeutic strategy

## Data availability statement

The original contributions presented in the study are included in the article/supplementary materials. Further inquiries can be directed to the corresponding author.

## Ethics statement

On December 16, 2019, the Institutional Review Board of the Lille University Hospital gave unrestricted approval for the anonymous use of all patients’ clinical, hormonal and ultrasound records (reference DEC20150715-0002). Written informed consent for participation was not required for this study in accordance with the national legislation and the institutional requirements.

## Author contributions

VS, PP and GR wrote the manuscript. CT wrote and performed biostatistics. All authors reviewed and approved the final version of the manuscript.
